# Hematopoietic Stem Cell Transplant for the Treatment of X-MAID

**DOI:** 10.3389/fped.2019.00170

**Published:** 2019-05-14

**Authors:** Sarah E. Henrickson, Isabelle Andre-Schmutz, Chantal Lagresle-Peyrou, Matthew A. Deardorff, Harumi Jyonouchi, Benedicte Neven, Nancy Bunin, Jennifer R. Heimall

**Affiliations:** ^1^Division of Allergy and Immunology, The Children's Hospital of Philadelphia, Philadelphia, PA, United States; ^2^Perelman School of Medicine, Institute for Immunology, University of Pennsylvania, Philadelphia, PA, United States; ^3^Laboratory of Human Lymphohematopoiesis, INSERM UMR 1163, Paris, France; ^4^Imagine Institute, Paris Descartes – Sorbonne Paris Cité University, Paris, France; ^5^The Children's Hospital of Philadelphia, Department of Human Genetics, Philadelphia, PA, United States; ^6^Division of Allergy/Immunology and Infectious Diseases, Rutgers-New Jersey Medical School, Newark, NJ, United States; ^7^Pediatric Immuno-Hematology Unit, Necker Children Hospital, Assistance-Publique Hopitaux de Paris, Paris, France; ^8^Division of Oncology, Bone Marrow Transplant and Cellular Therapy, The Children's Hospital of Philadelphia, Philadelphia, PA, United States

**Keywords:** SCID, HCT, WES, NBS, moesin

## Abstract

We report outcomes after hematopoietic stem cell transplant for three patients with X-MAID, including 1 patient from the originally described cohort and two brothers with positive TREC newborn screening for SCID who were found to have a T-B-NK+ SCID phenotype attributable to X-linked moesin associated immunodeficiency (X-MAID). A c.511C>T variant in moesin was identified via exome sequencing in the older of these siblings in the setting of low lymphocyte counts and poor proliferative responses consistent with SCID. He received reduced intensity conditioning due to CMV, and was transplanted with a T-depleted haploidentical (maternal) donor. His post-transplant course was complicated by hemolytic anemia, neutropenia, and sepsis. He had poor engraftment, requiring a 2nd transplant. His younger brother presented with the same clinical phenotype and was treated with umbilical cord blood transplant following myeloablative conditioning, has engrafted and is doing well. The third case also presented with severe lymphopenia in infancy, received a matched related bone marrow transplant following myeloablative conditioning, has engrafted and is doing well. These cases represent a novel manifestation of non-radiosensitive X-linked form of T-B-NK+ SCID that is able to be detected by TREC based newborn screening and effectively treated with HCT.

## Background

X-linked moesin-associated immunodeficiency (X-MAID) is a recently described form of primary immunodeficiency, characterized by severe leukopenia affecting T and B cells as well as neutrophils (1). X-MAID is one of a group of immunodeficiencies attributed to defects in cytoskeletal regulation (2). Moesin normally functions as an anchor of the actin filaments to the plasma membrane together with the proteins ezrin and radixin. These are collectively known as the ERM family of proteins. Lymphocytes have been described to have higher expression of moesin and ezrin as compared to radixin.

Prior to the publication of the X-MAID patient descriptions, isolated deficiency of moesin in mice was reported (3). The moesin deficient mice had an overall leukopenia with more pronounced loss of lymphocytes, particularly CD8 cells. In other mouse models, moesin deficiency is associated with a reduced proliferative capability of CD8 Treg cells and development of autoimmune symptoms consistent with lupus (3, 4). In addition, the mouse model demonstrated that the moesin deficient T and B cells demonstrated impaired ability to egress from the thymus and bone marrow, respectively, and that once in the periphery, moesin deficient T and B cells were also more likely to be held in the spleen and other secondary lymphoid organs. In X-MAID patients with the c.511C>T missense variant, moesin expression is reduced in B cells, CD4, and CD8 cells. These patients also demonstrated an impaired proliferative response to the phytohemaglutinin mitogen stimulation. In addition, a defect in thymic egress similar to these mouse models was described in the T cell populations of X-MAID patients bearing mutated moesin proteins (1). In addition, these patients were also noted to have neutropenia, the etiology of which is unclear. However, the carrier mothers of patients with the c.511C>T variants were noted to have skewed lionization in the peripheral blood mononuclear cell as well as polymorphonuclear cells. This skewing was not seen in their epithelial cells.

Despite their severely low T cell counts, only one of the seven originally described X-MAID patients was treated with allogeneic hematopoietic stem cell transplantation (HCT). Other, more well-known defects of cytoskeletal regulation associated with combined immunodeficiency include Wiskott Aldrich Syndrome (WAS) and DOCK8 deficiency (2). Both of these are able to be corrected though HCT. However, in WAS it has been noted that there is a spectrum of disease, and some patients do quite well with supportive care alone. In addition, WAS patients treated with HCT require a high degree of sustained engraftment to maintain correction of their immunodeficiency and to avoid development of severe autoimmunity (5). In contrast, Severe Combined Immune Deficiency (SCID) constitutes an unquestionable and urgent indication for HCT. Several studies have highlighted better outcomes for SCID patients diagnosed and transplanted early in life, a key element of justifying implementation of newborn SCID screening in the US and other countries (6, 7). However, the T-B-NK+ SCID phenotype poses unique challenges for transplant, since this phenotype can commonly be associated with radiosensitive SCID genotypes caused by *DCLRE1C* (Artemis), *LIG4, PRKDC, NHEJ1*, and *NBN* (NBS1) variants which can be associated with a higher risk of late effects when myeloablative conditioning is used (8). Prior to the description of X-MAID, only those SCID patients with pathologic variants in *RAG1* or *RAG2*, were non-radiosensitive forms of T-B-NK+ SCID and therefore able to better tolerate higher intensity conditioning than those with a similar phenotype due to radiosensitivity associated genetic defects.

## Case Presentations

We report here three cases of patients with X-MAID treated with HCT due to their initial SCID clinical presentation. The two first patients are Hispanic male siblings with X-MAID born to non-consanguineous parents and treated with HCT due to their initial clinical presentation being consistent with a phenotype of T-B-NK+ SCID. Written informed consent was obtained from the parents for publication of these cases and ongoing research based study of their children's immunodeficiency. Patient 1 was first identified due to abnormally low T cell receptor excision circles (TREC)s on newborn screening for SCID. There was no maternal engraftment and HIV testing was negative. There was no family history of SCID or other immunodeficiency. There was one maternal half-brother but no full siblings. The physical exam at presentation was normal. Prophylaxis was initiated at 1 week of life with IVIG and sulfamethoxazole/trimethoprim sulfate and fluconazole. He was confirmed to have T-B-NK+ SCID based on profound T and B cell lymphopenia (Table 1), and a very poor response to PHA stimulation (2% of normal). In addition he had neutropenia with absolute neutrophil count of 200 cells/uL. A commercially available comprehensive SCID genetic testing panel demonstrated him to be a carrier of a heterozygous pathogenic variant in *RMRP*, with neither an identified second variant nor any features of Cartilage-Hair Hypoplasia (OMIM 250250). He developed persistent neutropenia by 2 months of age (Table 1).

**Table 1:** Laboratory values for proband prior to and after mismatched related donor HCT.

	**Pre-HCT**	**Pre-HCT**	**3 months post HCT**	**6 months post HCT**	**7.5 months post HCT**	**8.5 months post HCT**	**12 months post HCT**	**16 months post HCT**	**2 years post HCT**
Age of the patient at analysis	DOL 5	2 months	5 months	8 months	9.5 months	10.5 months	14 months	18 months	27–28 months
ANC/uL	1,600	200	4,940	1,020	396^*^	310^*^	320^*^	3,630^*^	670
ALC/uL	200	536	810	156	382	640	1,620	1,650	1,460
CD3+/ul	41	175	55	77	184	421	1,281	1,524	1,172
CD4+/uL	28	48	5	12	84	135	282		413
CD8+/uL	11	123	36	50	81	241	847		694
CD4+/CD45RA+ (%)		5.7	0.4	2.9	9.5	10.6	6.2		11.7
CD4+/CD45RO+ (%)		5.4	0.6	5.7	10.2	9.9	10.9		16.1
Mitogens (PHA) % of control CPM		2% CPM 887	< 1% CPM 1,127		1% CPM 2,439				
TRECS^***^				1,398		2,283			2,962
CD19+ B cells/uL	3	255	704	46	167	84	86		91
NK cells/uL	78	110	51	32	36	141	245		164
Chimerism^∧∧^			82%	26%	25%		26%	41%	
Chimerism (CD3)			57%	36%	76%		83%	83%	
Chimerism (CD19)			96%	49%				15%	
Chimerism (NK)			96%	70%					
Chimerism (Myeloid)			79%	17%				2%	

* , on G-CSF supplementation;

*** , Count per 10^6^ CD3 cells;

∧∧ *, Unenriched whole blood*.

Diarrhea developed at 2 months, and he was noted to be CMV PCR positive in blood. Therapy was initiated with valganciclovir. There was no suitable HLA-matched unrelated donor or cord blood and he emergently received a maternal haploidentical CD3/CD19-depleted HCT 2 weeks later (Cell dose: CD34 11.6 × 10^6^ cells/kg; CD3 and CD19 below level of detection). Due to a lack of identification of a known *RAG1* or *RAG2* variant and without results demonstrating lack of radiosensitive SCID in the setting of a T-B-NK+ phenotype, the decision was made to minimize alkylator therapy. He received reduced intensity conditioning that included thymoglobulin (3 mg/kg daily for three doses) and busulfan (1 mg/kg for eight doses with pharmacokinetics, PK 1159 micromole^*^min/L). He became CMV PCR-negative 2 weeks after transplant. At discharge, he remained on fluconazole and pentamidine prophylaxis, IVIG, and treatment-dose foscarnet. He developed autoimmune hemolytic anemia (AIHA) at 3 months post-transplant, which was treated with methylprednisone. At 5 and 7 months post-transplant he was admitted with *Enterobacter* septicemia associated with his central line. He developed neutropenia at 6 months post HCT that was progressive, but responsive to G-CSF supplementation 3 days a week to maintain ANC over 500. At 16 months post HCT, his T cell engraftment was 83% and CD3 counts were normal, but TREC counts remained low, with absent myeloid engraftment, G-CSF dependent neutropenia, poor B cell counts with low IgA and IgM and continued need for immunoglobulin replacement. Bone marrow biopsy demonstrated normocellular marrow with tri-lineage hematopoiesis. CMV PCR remained negative throughout this period of neutropenia.

At 3 years, 2.5 months of age, he received a second MMRD HCT using TCR alpha/beta depleted PBSC from the same donor, in the setting of no other available match. Conditioning was myeloablative and consisted of fludaribine (40 mg/m^2^ for 4 doses), melphalan (140 mg/m^2^ for 1 dose), thiotepa (10 mg/kg for 1 dose) and thymoglobulin (3 mg/kg for 3 doses) with a goal to correct his incomplete immune reconstitution, and achieve myeloid engraftment. He demonstrated recovery of neutrophil counts to >1,000 cells/uL by 10 days post HCT with 100% engraftment at 3 months post-transplant. He did experience reactivation of CMV which was resistant to treatment with foscarnet, but did respond to CMV-specific viral cytotoxic T cells. Unfortunately, at 3 months post-transplant he developed polyserositis of unclear etiology which manifested as pericardial and pleural effusions. His symptoms progressed to ARDS and he had acute pulmonary hemorrhage which led to his death.

Clinical exome sequencing of the proband and the parents as a trio analysis was performed 6 months after his first transplant utilizing DNA obtained pre-transplant from a skin biopsy. He was noted to have a heterozygous variant of undetermined significance (VUS) in *PRKDC* (c.1945T>C; Table 2). Deletion and duplication testing of *PRKDC* identified no second variant, making it unlikely that this variant was causal for an autosomal recessive form of T-B-NK+ radiosensitive SCID. In contrast, a VUS in *MSN*, an X-linked gene encoding moesin (c.511 C>T; Table 2) was identified and confirmed by Sanger sequencing. This variant was reported based on moesin-deficient murine data showing decreases in B and T cells (3). This variant was not observed in the ExAC dataset (exac.broadinstitute.org), where *MSN* also demonstrates a pLI of 0.99 and a missense constraint z-score of 2.78, indicating poor tolerance for loss or altered function of this gene. In addition, this variant was later reported to be associated with X-MAID.

**Table 2:** Immune variants on proband WES.

**Gene**	**Chr: position, ref, alt**	**CADD score**	**PROVEAN score**	**Depth of coverage**
MSN	X: 64951012 C>T pR171W	33	−6.88	54x
PRKDC	ch8: 48842520 T>C pF649L	20.3	−3.42	107x

The second affected boy, a full sibling to the proband, also presented with a presumptive positive TREC screen on NBS for SCID, with flow cytometry demonstrating a virtual absence of T, B and NK cells and poor proliferation to PHA stimulation but normal neutrophils. At 1 week of life he was started on sulfamethoxazole/trimethoprim sulfate, fluconazole prophylaxis and IVIG (Table 3). Due to the known variant in his brother, patient 2 underwent targeted Sanger sequencing for the moesin variant and he was confirmed to have the same pathogenic variant in moesin as his older sibling (Table 2). Although he did not have CMV infection, after discussion with the family and care team, it was decided that this patient would also be treated with HCT for his T-B-NK+ SCID phenotype. At 2 months, he underwent HCT with a 6/6 matched cord blood (Cell dose: CD34 3.59 × 10^5^ cells/kg; CD3 3.75 × 10^5^ cells/kg) with conditioning that included busulfan (0.8 mg/kg for 16 doses with pharmacokinetics for AUC: PK 1526 micromole^*^min/L), fludarabine (1.33 mg/kg for 4 doses), and thymoglobulin (3 mg/kg daily for 3 doses). He was also found to have congenital hypothyroidism on NBS and was started on levothyroxine.

**Table 3:** Laboratory values for younger sibling prior to and after matched cord blood HCT.

	**Pre-HCT**	**39 days post HCT**	**81 days post HCT**	**1 year post HCT**	**2 years post HCT**
Age of the patient at analysis	DOL7	3.5 months of age	5 months of age	13 months of age	26 months of age
ANC/uL	2,020	4,620	5,160	10,320	1,630
ALC/uL	170	600	1,320	450	3,210
CD3+/ul	136	232	182	550	2,087
CD4+/uL	103		164	277	1,197
CD8+/uL	33		11	181	745
CD4+/CD45RA+ (%)	39.4		2	6.2	28.4
CD4+/CD45RO+ (%)	16.7		12.4	29.8	8.4
Mitogens (PHA) % of control CPM	2% CPM 5,938		4% CPM 7,747		100% CPM 171,606
TRECS^***^			148 (1,484–5,327)	4,578 (>6,794)	25,195 (>6,794)
CD19+ B cells/uL	10		558	0	870
NK cells/uL	24			213	173
Chimerism^∧∧^		100%	100%	100%	100%
Chimerism (CD3)		QNS	QNS	100%	100%
Chimerism (CD19)		QNS	QNS	100%	100%
Chimerism (NK)		100%	100%	100%	100%
Chimerism (Myeloid)		100%	100%	100%	100%
TCR Vb spectratyping				Majority oligoclonal	

*** , Count per 10^6^ CD3 cells;

∧∧ *, Unenriched whole blood*.

Similarly to his older brother, the post-transplant course was complicated by AIHA, and treated with rituximab and prednisone. He had limited skin graft vs. host disease (GVHD) and was treated with sirolimus. Post HCT infections included bacteremia with a central line and rhinovirus. In contrast to his brother, his engraftment is 100% across all lineages, with normal T cell numbers, normal TREC counts and normal proliferative response to PHA at 2 years post HCT. He has a normal ANC without need for G-CSF (Table 3). He did experience initial rituximab-induced B cell aplasia and reduced IgA and IgM production, but this has recently improved with normal IgA and IgM at the 24 month post HCT time point.

Patent 3 corresponds to Patient 4 of the initial published cohort of patients with X-MAID (1) (Table 4). He presented with an early onset and persistent lymphopenia and fluctuating neutropenia which responded to G-CSF treatment discovered during the exploration of an ulcerating ecthyma. Hemizygous variant in MSN gene (c.511 C>T) was found by targeted Sanger sequencing. T cells responded poorly to PHA stimulation (Table 4). At 1 year of age, he developed varicella requiring hospitalization and treatment by Varicella Zoster Immunoglobulin (VZIg) and acyclovir. Due to low T cell counts, at 5 years of age he received a matched related HCT from his geno-identical sister utilizing bone marrow (3.8 × 10^8^ WBC/kg; 3.8 × 10^6^ CD34+cells/kg, 29.6 × 10^6^ CD3+cells/kg). At that time he was in good general health, with no infectious problem. A myeloablative conditioning regimen was used (Busulfan 20 mg/kg /Cyclophosphamide 200 mg/kg) and Cyclosporine as prophylactic treatment of GVHD. He developed skin grade 2 acute GVHD at day 10 post HCT that was resolved by cyclosporine and prednisone (5 mg/kg/day) and that recurred in ahepatic and digestive moderate form before complete resolution with corticotherapy and cyclosporine. He experienced acute pancreatitis day 26 post HCT, which was treated with octreotide, morphine, and tiemonium methysulphate. He experienced Varicella zoster 3 months post HCT treated by acyclovir. Chimerism has consistently been 100% of donor. He is in complete remission of his disease, with normal numbers of neutrophils and lymphocytes.

**Table 4:** Laboratory values for patient 3 before and after matched sibling HCT.

	**Pre-HCT**	**Pre-HCT**	**2 months post HCT**	**15 years post HCT**
Age of the patient at analysis	12 months	56 months	65 months	20 years
ANC/uL	949	200	7,500	4,850
ALC/uL	586	700	400	
CD3+/ul		658	276	1,548
CD4+/uL		203	60	720
CD8+/uL		469	172	285
CD4+/CD45RA+ (%)		33		
CD4+/CD45RO+ (%)		86		
Mitogens (PHA) % of control CPM		11%		
CD19+B cells/uL		7	0	
NK cells/uL		56	Not tested	
Chimerism^∧∧^			100%	100% @ 6 years post HCT

∧∧ *, Unenriched whole blood*.

## Discussion

Subsequent to the identification of the proband's pathogenic variant, 7 male patients were described with moesin-deficient (X-MAID), with 6 of 7 having the same *MSN* variant as noted in our family (1). While all of these patients demonstrated reduced T and B cells as well and hypogammaglobulinemia, there was significant variability in the initial clinical presentation. Of the 7 patients, 6 presented with Varicella in childhood, causing pneumonitis in 3 patients. Skin manifestations were common with 5 of the 7 noted to have eczema and 3 of the 7 with severe Molluscum contagiosum. Others presented with recurrent GI, GU, and respiratory infections. Six out of seven patients have been managed with prophylactic antibiotic treatment and immunoglobulin replacement and, in one case, chronic G-CSF for neutropenia. There have been two additional cases of X-MAID published (9, 10). In one of these, similar to ours, profound lymphopenia was detected in follow up testing to an abnormal SCID NBS (9). This then led to WES and detection of the same p. R171W variant at 25 months of age. However, in contrast to our cases, this patient's mitogen proliferation response to PHA was robust, his clinical infection history was mild to age 2 years, and therefore he was managed supportively rather than with HCT. Of note this child was also noted to have significant eczema, a feature that has been described in other diseases of cytoskeletal regulation. Another group described detection of X-MAID due to the p. R171W variant via WES performed in a young adult man who had experienced lifelong signs and symptoms of combined immunodeficiency, including lymphopenia and neutropenia with recurrent Varicella and bacterial infections in early childhood (10). This patient had experienced clinical improvement in stability with a decrease in infection frequency following initiation of G-CSF and immunoglobulin replacement as supportive therapies. The growing body of literature describing patients with the same variants in *MSN* gene reveals variable clinical manifestations. The reason for this is not yet elucidated, but, as previously described for other genetic forms of SCID, environmental factors could be involved in the phenotypic heterogeneity (11).

We present these three cases to demonstrate that X-MAID patients detected by either Sanger sequencing (for the oldest one) or via TREC-based newborn screening programs for SCID can present in infancy with a phenotype of T-B-NK+ SCID which benefits from correction with HCT. This represents an additional non-radiosensitive form of T-B- NK+ SCID caused by X-linked inheritance. While this is a limited report, the use of a fully matched cord or genoidentical donor and myeloablative conditioning was associated with myeloid engraftment and more robust immune reconstitution.

## Conclusion

In this presentation of 3 X-MAID patients presenting with a SCID phenotype, HCT with myeloablative conditioning and utilizing a fully matched blood donors led to a better outcome than MMRD with reduced intensity conditioning.

X-MAID patients appear to have a spectrum of clinical severity despite the presence of identical variants. The reason for this is unclear, and this should be studied in greater depth through international registry collaboration with detailed immunoprofiling and mechanistic studies.

## Data Availability

All relevant data is contained within the manuscript.

## Ethics Statement

This study was carried out in accordance with the recommendations of the CHOP IRB with written informed consent from all subjects. All subjects gave written informed consent in accordance with the Declaration of Helsinki. The protocol was approved by the CHOP IRB.

## Author Contributions

SH and JH wrote the paper. All authors reviewed and edited the paper. IA-S and CL-P shared data on X-MAID patients prior to publication of their case series to confirm the effect of the familial mutation on MSN function. SH, MD, HJ, BN, NB, and JH cared for the patients clinically.

### Conflict of Interest Statement

SH participated in two *ad hoc* advisory boards for Horizon Pharma in 2017. The remaining authors declare that the research was conducted in the absence of any commercial or financial relationships that could be construed as a potential conflict of interest.
